# Module for SWC neuron morphology file validation and correction enabled for high throughput batch processing

**DOI:** 10.1371/journal.pone.0228091

**Published:** 2020-01-23

**Authors:** Damien M. O’Halloran

**Affiliations:** Department of Biological Sciences, The George Washington University, Washington D.C., United States of America; Augusta University, UNITED STATES

## Abstract

SWC files are a widely used format to store neuron morphologies, and are used to share digitally reconstructed neurons using NeuroMorpho.org as well as predict functional attributes using simulation environments such as NEURON. Here we set out to develop an easily accessible tool to validate and correct SWC formatted files with an emphasis on high throughput batch processing. *SWC_BATCH_CHECK* is a package that provides a suite of methods to parse and correct the syntactic structure of a directory of SWC files. This tool ensures that user specified structures such as the soma or basal dendrite are correctly connected while fixing morphological features. This tool will report on missing or invalid data values while also returning basic statistical features for each file. *SWC_BATCH_CHECK* was validated and tested using thousands of individual SWC files to benchmark runtime performance and efficacy in both reporting on and correcting disparate SWC file features. *SWC_BATCH_CHECK* is open source and freely available to all users without restriction with guidelines and requirements provided to ensure straightforward installation and execution.

## Introduction

In order to detail the intricate structure of neurons, and even map the overlying circuitry, researchers can trace the morphology using light microscopy and use microscopic images to digitally reconstruct neuronal organization and arrangement. These reconstructions are then typically stored and shared using the SWC neuron morphology format [1,2]. The SWC file is composed of header entries and data entries. The data line entries are organized by structure e.g. soma, coordinates, radius, and the parent connection. The resulting data structure is a tree-like set of nodes where each node has a set of 3-dimensional coordinates and a radius, and each tree (or neuron) having a single root node defined at the soma. SWC file formats can be uploaded and shared using NeuroMorpho.org [3], which houses over 100,000 digital reconstructions, and then used to predict circuit function and output using simulation environments such as NEURON [4–6]. Because of the increasing scale and complexity of available circuits, we here set out to develop a simple tool that can validate and report on SWC file format in a high-throughput manner. Quality control for SWC formatted files is imperative in order to maintain morphological integrity and to avoid downstream artefacts. Furthermore, SWC files have now become widely used in cloud-based downstream applications and pipelines (e.g. https://www.nsgportal.org), and so diverse preprocessing tools are necessary to programmatically handle SWC file validation.

## Methods

*SWC_BATCH_CHECK* was developed using Perl 5 and successfully tested on Microsoft Windows 7 Enterprise ver.6.1, Linux Mint ver. 19 Tara, and MacOSX Mojave ver.10.14.5. *SWC_BATCH_CHECK* is freely available as a GitHub repository: https://github.com/dohalloran/SWC_BATCH_CHECK. The module’s main library is composed of a single package—*SWC_BATCH_CHECK*.*pm*—and all dependencies are available through CPAN (https://metacpan.org/) which makes the module very straight forward to implement. *SWC_BATCH_CHECK* is written using modern Perl best practices and instantiated by collecting user arguments from a Moose class (https://metacpan.org/pod/Moose). Sample data are provided at the GitHub repository and directions for installation and execution are described in the README.md file. The only required argument to run *SWC_BATCH_CHECK* is the path to a directory containing SWC file(s), which is supplied using the --*d* flag. The program will generate a new folder in the current working directory to output corrected SWC files, as well as an error log that details inconsistencies and errors within each file by describing the issue and the data line containing the problem. Warnings detected include incorrect indexing; no root node; file not starting at the soma; as well as missing data values. The user can also supply one or more structures (e.g. soma, axon, basal dendrite (*basal* flag), or apical dendrite (*apic* flag)) as arguments and the program will make corrections to the specified structure(s). In the case of dendritic structures, basal dendrites should only connect to more basal dendrites or the soma, and if an incorrectly connected dendrite is identified it will automatically correct this defect providing the subsequent node is correctly connected. The same logic is applied for apical dendrite structures. Branch compartments in which the radius is set to zero, are changed to the parent size by supplying the --*rad* flag argument. Other corrections and warnings are detailed on the GitHub repository. In addition, the program will also return basic statistics for each SWC file within the supplied directory as well as the command line arguments used. This feature can be piped to a file and used for later troubleshooting.

## Results and discussion

**Fig 1:**
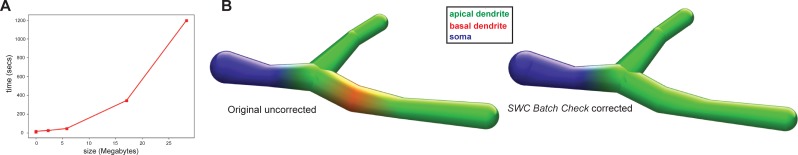
Runtime performance testing of *SWC_BATCH_CHECK*. **(A)** Directories containing different numbers of SWC formatted files were provided as input and ran using default settings. Directory sizes were: 0.000285MB, 0.000627MB, 2.320682MB, 5.773404MB, 17.037202MB, and 28.301MB. From this analysis the equation of the regression line was *y* = 2.631 + 0.023⋅*x;* correlation coefficient, *r* = 0.9469. **(B)** Validation testing of uncorrected SWC file containing an incorrectly connected basal and apical dendrite (left image) and a SWC file corrected using *SWC_BATCH_CHECK* (right image) that fixes the inconsistency of apical to basal connection. Images were rendered using *SharkViewer* [12].

**Fig 2:**
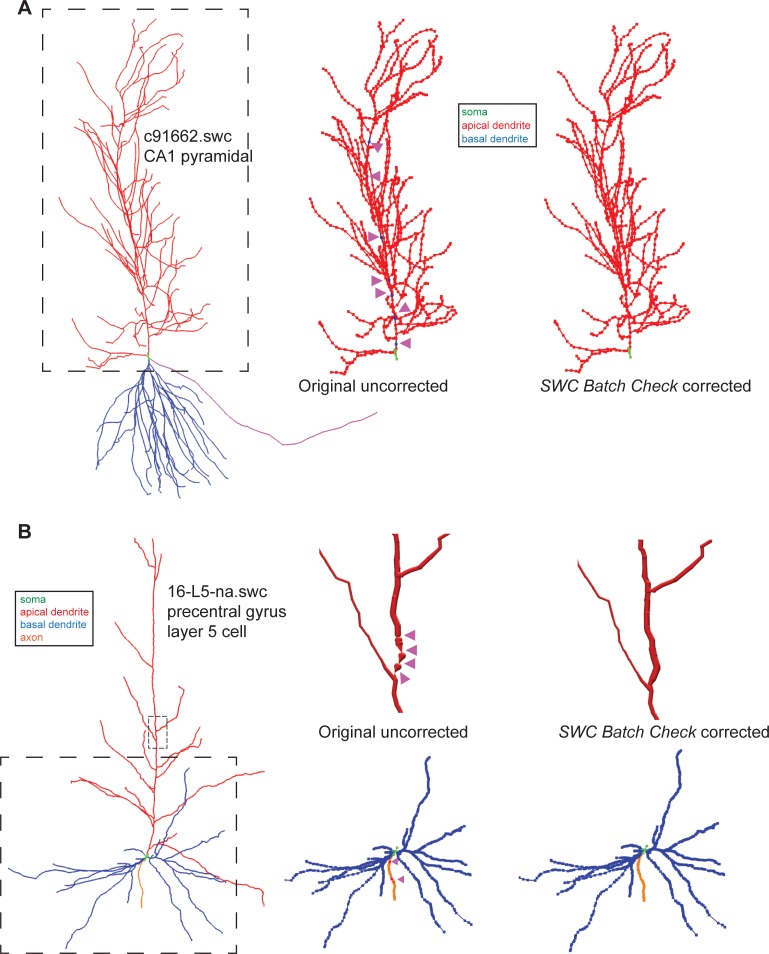
Testing *SWC_BATCH_CHECK* on real neuron reconstructions from *NeuroMorpho*. **(A)** Full reconstruction of a CA1 pyramidal cell (*NeuroMorpho* ID NMO_00227; neuron name c91662) is shown in the leftmost image. Errors were introduced (arrowheads) to insert basal dendrite into the apical dendrite region (middle image). The apical region from the corrected output file using *SWC_BATCH_CHECK* with the correct apical dendrite syntactic structure is rendered in the rightmost image. **(B)** Reconstruction of a precentral gyrus layer 5 pyramidal cell (*NeuroMorpho* ID NMO_05515; neuron name 16-L5-na) is shown in the leftmost image. The radius was changed to zero for several compartments along the primary apical branch (upper middle image, arrowheads denote errors). The region containing these errors is shown in the full reconstruction (leftmost image) as a small dashed rectangle. Part of the corrected SWC file using *SWC_BATCH_CHECK* is shown as the rightmost upper image. Errors were also introduced into the axonal region (marked by arrowheads) of the cell by changing several axonal compartments to apical dendrite syntax (middle lower image). The resulting SWC file from *SWC_BATCH_CHECK* was visually rendered in rightmost lower image. For all images green indicates soma, red indicates apical dendrite, blue for basal dendrite, and orange for axon. SWC files for all images were rendered using neuTube [9] except for the upper images in **(B)** which were generated using *SharkViewer* [12].

In order to validate and test *SWC_BATCH_CHECK* we first benchmarked runtime performance by examining execution speed as a function of file size (Fig 1A). Directories comprised of SWC formatted data files that varied from a few hundred KB up to over 28 MB in size were used to benchmark the runtime performance using default settings. In each case, *SWC_BATCH_CHECK* efficiently returned summary statistics and validated each file correctly. The runtime will scale with the number of methods called, and for reference a typical SWC file for a single neuron is ~60 KB and therefore the upper limits of our runtime analysis contained over 500 individual SWC files. In order to validate and test the corrections and warnings returned by *SWC_BATCH_CHECK* we performed validation testing on thousands of SWC files to ensure appropriate reporting. Some of these SWC files used in validation are available in the sample_input folder within the GitHub repository. In Fig 1B, we show an example correction that identifies a basal dendrite incorrectly connected to an apical dendrite (left image), and after running this SWC file through *SWC_BATCH_CHECK* the resulting corrected SWC file fixes this error by changing the inconsistent structure to an apical dendrite (right image). We also include examples from real neuron reconstructions in Fig 2. In Fig 2A, a CA1 hippocampal pyramidal cell was used. The *NeuroMorpho* ID for this cell is NMO_00227 and neuron name is c91662. The cell was reconstructed from a female Sprague-Dawley rat [7]. The full reconstruction is depicted in Fig 2A (leftmost image). For demonstration purposes, we introduced errors into the SWC file to insert basal dendrite compartments within the apical dendrite branch (Fig 2A middle image, errors denoted by arrowheads). This erroneous SWC file was provided as input to *SWC_BATCH_CHECK* using the --*basal* and --*apic* flags and the apical region from the resulting SWC file was visually rendered with the correct apical dendrite syntactic structure (Fig 2A, rightmost image). In Fig 2B, we used the SWC file digital reconstruction of a precentral gyrus layer 5 principal pyramidal cell from the frontal neocortex of a male Sprague-Dawley rate [8]. The *NeuroMorpho* ID for this file is NMO_05515 and the neuron name is 16-L5-na. A visual rendering of the full cell is shown in Fig 2B (leftmost image). In this case, we introduced two different types of errors into the underling SWC file. Firstly, we changed the radius to zero for several compartments along the primary apical branch. This region is shown in Fig 2B (leftmost image) as a small dashed rectangle; in the upper middle image of Fig 2B, this region is magnified to visually depict the effect of introducing zero radius compartments, which are marked by arrowheads. This modified SWC file was then supplied as input to *SWC_BATCH_CHECK* using the --*rad* flag and part of the resulting SWC file is shown as the rightmost upper image in Fig 2B. Next, we introduced errors into the axon region of the cell by changing several axonal compartments to apical dendrite syntax instead. These regions are denoted by arrowheads in the lower middle image of Fig 2B; the corresponding field of view is denoted in the leftmost whole image as a large dashed box. The SWC file containing these errors was used as input for *SWC_BATCH_CHECK* using the --*axon* flag and the basal region from the resulting output SWC file is visually rendered in Fig 2B (rightmost lower image). In each of the examples described above, *SWC_BATCH_CHECK* correctly documented all the errors that were introduced and made the appropriate corrections in the resulting output SWC file. In all cases, it is best to first examine the error log to understand as many putative errors as possible, and then use various flags to correct these errors.

Finally, it is important to recognize that multiple tools are available that perform some or all of the functions described within *SWC_BATCH_CHECK* [9–11]. One of the main distinctions between *SWC_BATCH_CHECK* and some other tools is that other SWC editors include visual rendering capability; for example, neuTube [9] allows the user to visually inspect the structural integrity of neuron reconstructions and edit branches. While this is a limitation of *SWC_BATCH_CHECK*, it was not the goal of developing the module described here, rather the goal was to produce an intuitive module to automate the task of identifying errors in digital neuronal reconstructions in SWC format. In developing this tool, the major goals were: accessibility, ease of use, documentation, as well as applicability of options for large-scale analyses. In summary, I here describe a lightweight Perl package for robust analysis, validation, and correction of SWC neuron morphology files batches, which is intuitive, easily installed, and freely available on GitHub at: https://github.com/dohalloran/SWC_BATCH_CHECK

### Availability of data and materials

- Project name: SWC_BATCH_CHECK
- Project home page: https://github.com/dohalloran/SWC_BATCH_CHECK
- Operating system(s): Platform independent
- Programming language: Perl
- Other requirements: none
- License: GNU
- Any restrictions to use by non-academics: no restrictions or login requirements

## Abbreviations

KB: Kilobytes
MB: Megabytes
CPAN: Comprehensive Perl Archive Network
